# MAGENTA (Making Genetic testing accessible): a prospective randomized controlled trial comparing online genetic education and telephone genetic counseling for hereditary cancer genetic testing

**DOI:** 10.1186/s12885-019-5868-x

**Published:** 2019-07-02

**Authors:** Nadine Rayes, Deborah J. Bowen, Tara Coffin, Denise Nebgen, Christine Peterson, Mark F. Munsell, Kathleen Gavin, Rebecca Lechner, Jamie Crase, Deborah Polinsky, Iris Romero, Stephanie V. Blank, Douglas A. Levine, Barbara M. Norquist, Elizabeth M. Swisher, Karen H. Lu

**Affiliations:** 1 University of Texas MD Anderson Cancer Center, Houston, TX 77030 USA; 2 University of Washington, Seattle, Washington 98195 USA; 3 Minnesota Ovarian Cancer Alliance, Minneapolis, MN 55407 USA; 4 Patient Advocate, Mercer Island, WA 98040 USA; 5 Facing our Risk of Cancer Empowered, Tampa, Florida 33647 USA; 6 University of Chicago, Chicago, IL 60637 USA; 7 Icahn School of Medicine at Mount Sinai, New York, NY 10029 USA; 8 New York University, New York, NY 10016 USA

**Keywords:** Genetic testing, Genetic counseling, Hereditary cancer, Ovarian cancer

## Abstract

**Background:**

Studies have consistently indicated that the majority of individuals meeting the US Prevention Services Task Force guidelines for genetic testing have not had genetic counseling or testing. Despite increased availability and lower costs of multiplex cancer gene panels, there remains a gap in genetics services that has not been addressed by the current care delivery models. Lower cost of DNA sequencing with online patient-initiated ordering could increase test availability, but the ideal quantity and delivery method of patient education is not known. We hypothesized that online genetic education and testing with access to board certified genetic counselors could improve access to genetic testing while maintaining test quality and clinical utility. The MAGENTA (MAking GENetic Testing Accessible) trial is a nationwide randomized study designed to compare the effectiveness of online genetic education with pre- and post-test telephone genetic counseling to three potentially more accessible alternative approaches: online genetic education with optional telephone counseling, online genetic education with required pre-test telephone genetic counseling, and online genetic education with required post-test telephone genetic counseling.

**Methods:**

3000 women nationwide will undergo genetic testing for 19 hereditary cancer genes. This is a randomized four-arm non-inferiority study with equal randomization. The four study arms were selected to independently assess the delivery of genetic information both before and after genetic testing (pre-test and post-test) by either requiring telephone genetic counseling or providing only online education with optional telephone counseling. Patients have post-test telephone counseling when testing positive for a pathogenic inherited mutation in all four arms. Surveys measuring psychological, behavioral and cognitive state are completed online at baseline, 3 months, 12 months and 24 months post-results disclosure. The primary study outcome is cancer-risk distress at 3 months post-result disclosure.

**Discussion:**

This trial will assess the use of a genetic service model using online access and electronic education, while evaluating the need for personal pre- and post-test genetic counseling. Data from this study may lead to increased options for delivery of genetic testing and possibly increase access to genetic testing. Identifying more individuals with inherited cancer susceptibility will allow targeted cancer prevention.

**Trial registration:**

Clinicaltrials.gov: NCT02993068 (registered December 14, 2016).

## Background

Rapid developments in cancer genetics over the past decade have changed the landscape of genetic testing for hereditary cancer. The availability of next generation sequencing, coupled with the Supreme Court decision invalidating patents on human genes, has led to substantially decreased cost of germline testing and has created a shift from single syndrome or gene by gene testing to multiplex gene panel testing. Identification of individuals who carry a mutation in cancer predisposition genes such as *BRCA1* and *BRCA2* allows for prevention and early detection.

However, studies have consistently indicated that the majority of individuals meeting the United States Prevention Services Task Force (USPSTF) guidelines for genetic testing have not had genetic counseling or testing [1]. Childers and colleagues estimate that over 1.2 million women with a history of breast and/or ovarian cancer have not undergone genetic testing despite meeting evidence-based guidelines for testing [2]. Cascade testing is the process of testing biological relatives of individuals who have been identified to have a genetic mutation. Cascade testing for inherited cancer risk has been identified as a public health priority by the Centers for Disease Control and Prevention (CDC), but uptake of cascade testing is low, with only 30% of eligible first-degree relatives receiving testing [3]. These findings indicate that despite increased quality and lower costs of multiplex cancer gene panels, there remain important barriers to genetic testing.

Various service delivery models (SDMs) have been explored and adopted to increase access to cancer genetic testing services. The traditional SDM for cancer genetic testing employs in-person pre-test and post-test genetic counseling. Telephone genetic counseling has been shown to be non-inferior to in-person counseling and is now a widely accepted SDM with the potential to provide services to patients who live far from genetics specialists [4, 5]. Online genetic testing services are now commercially available but the amount, type and degree of personalization of paired education and counseling required in order to optimize patient understanding and minimize distress is unknown.

### Proposed alternate remote testing service delivery model

Online access to physician ordered genetic testing for clinically actionable hereditary cancer genes has the potential to increase access to genetic testing while maintaining test quality and clinical utility. Smit and colleagues found that individuals believe online communication is an acceptable mode of receiving medical information, and that it is important to have a health professional available to discuss questions and content as needed [6]. One study has examined the use of online genetic testing for cascade testing of family members and found it to be an effective approach to implementing cascade testing, citing an uptake of cascade testing in 47.5% of first degree relatives [7].

The availability of online genetic education detailing the benefits and limitations of hereditary cancer testing with access to board certified genetic counselors offers a promising alternative that may overcome barriers faced by current SDMs. Such a model could allow both genetics and non-genetics clinicians to order genetic testing while providing patients with detailed online information regarding hereditary cancer testing, overcoming the major barriers of physician coordinated testing, including limitations of physician time and knowledge. Remote access testing may eliminate the major barriers of in-person counseling, including time and money spent on travel, while increasing flexibility and accessibility, which continue to be challenges of both in-person counseling and telephone counseling. Remote access genetic testing via the use of an online genetic services platform allows individuals to access information using the internet with the ability to revisit if they choose, avoiding the need for appointments and allowing for patient-driven and convenient genetic services. Electronic copies of genetic test results may be shared easily with family members and medical providers. For individuals found to have a pathogenic mutation, online platforms facilitate easy information sharing, and the option of online access to testing services may promote the uptake of cascade testing in blood relatives.

One question in this proposed SDM is how much personalized counseling is needed both pre-test and post-test to optimize patient outcomes. Does required personalized counseling reduce patient distress and improve understanding or does it add unnecessary cost and time and reduce likelihood of test completion? Is pre- or post-test counseling more important and if counseling is provided as an option, how many patients will utilize telephone counseling, and can we identify patient subsets who have greater benefit from personalized counseling? To address these considerations, we are conducting a prospective study to evaluate different amount of personalized genetic counseling within an online genetic testing model to determine less counseling is non-inferior to our current accepted SDM at delivering hereditary cancer risk information. The purpose of this paper is to describe the design of the MAGENTA study, a prospective randomized trial of online genetic education vs telephone genetic counseling for hereditary cancer genetic testing.

## Methods/design

### Study aim and hypothesis

The MAGENTA (MAking GENetic Testing Accessible) trial is designed to compare the effectiveness of pre- and post-test telephone counseling to three potentially more accessible alternatives for individuals undergoing analysis of 19 hereditary breast and ovarian cancer (OC) genes. The genes included are: *ATM, BARD1, BRCA1, BRCA2, BRIP1, CDH1, CHEK2, EPCAM, MSH2, MSH6, MLH1, NBN, PALB2, PMS2, PTEN, RAD51C, RAD51D, TP53,* and *STK11*. Our primary hypothesis is that the outcomes for subjects receiving one of the three alternative approaches utilizing genetic education online will not be inferior to those of participants receiving a current acceptable standard of telephone genetic counseling both pre- and post-testing [4, 5].

### Study design

**Fig. 1:**
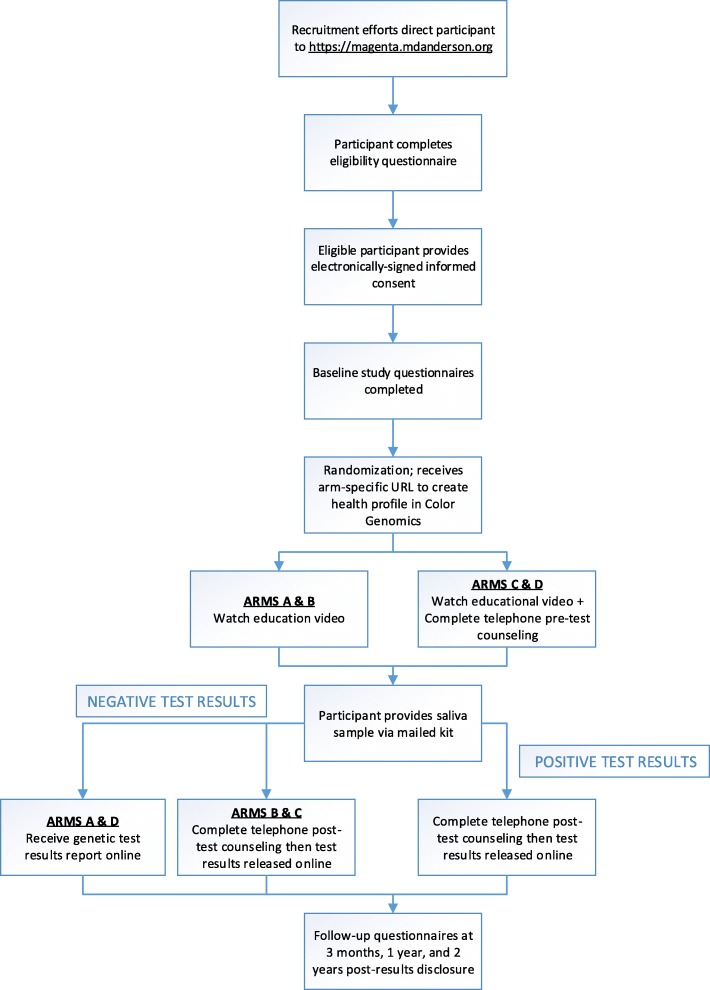
MAGENTA study schema

After online eligibility screening and electronic study consent (as approved by the Human Subjects Division of the MD Anderson Institutional Review Board), randomization into one of four study arms is performed automatically using a web-based data management platform [8]. The four study arms were selected to independently assess the delivery of genetic information both before and after genetic testing (pre-test and post-test) by either telephone genetic counseling or online education alone. The control arm requires mandatory pre and post-test telephone counseling while the other three arms provide various combinations (Table 1). Participants are mailed a saliva collection kit after all pre-test online education and/or genetic counseling is completed, based on study arm. Analysis of 19 OC genes is completed once the participant returns the saliva sample. As an additional safety measure, any participant found to carry a pathogenic or likely pathogenic mutation has required post-test telephone counseling and any participant desiring genetic counseling is provided with telephone genetic counseling regardless of study arm. A study schema can be found in Fig. 1. Participants were stratified into one of two risk groups: individuals with a personal and/or family history of cancer, and those with a known familial mutation (cascade group).

**Table 1:** Study Arms

	Pre- Genetic Testing	Post- Genetic Testing
Arm A	Online educational video	Online Test Results Report
Arm B	Online educational video	Online Test Results Report + Telephone Genetic Counseling
Arm C (Control)	Online educational video + Telephone Genetic Counseling	Online Test Results Report + Telephone Genetic Counseling
Arm D	Online educational video + Telephone Genetic Counseling	Online Test Results Report

Note: optional telephone genetic counseling is allowed at any point in all arms at patient request and is mandated post-test for all patients with pathogenic mutation regardless of study arm

### Study population

The MAGENTA study’s target population is women that may be at increased risk to develop ovarian cancer based on their personal or family history of cancer or due to a known cancer susceptibility gene mutation in the family. Women must be 30 years of age or older and must have at least one ovary, as we hope to identify women who will be able to take actionable measures to reduce ovarian cancer risk. As a safety precaution, all participants are required to identify a local healthcare provider with whom their genetic test results will be shared. Complete eligibility criteria are outlined in Table 2. Approximately 5200 women will be enrolled with a goal of 3000 completing genetic testing. We estimate that we will need to screen between 7000 and 10,000 women using an online eligibility questionnaire.

**Table 2:** Eligibility Criteria

*Inclusion Criteria*	
*Participants must meet each of the following four criteria*	
1) Women ages 30 or older	
2) Have access to a healthcare provider and be willing to share genetic results with that provider	
3) Have at least one ovary	
4) Have a valid United States mailing address for receipt of saliva kit	
*Additionally, participants must also meet any one of the following six criteria:*	
5) Diagnosed with breast cancer at age 45 or younger	
6) Diagnosed with triple negative breast cancer at age 60 or younger	
7) Have one biological relative with a mutation in BRCA1, BRCA2, BRIP1, PALB2, RAD51C, RAD51D,	
BARD1, MSH2, MSH6, MLH1, or PMS2	
6) Have a biological relative with ovarian cancer	
7) Have at least 2 biological relatives with breast cancer on the same side of the family, one of which is < age 50	
8) Have one biological male relative with breast cancer	
*Exclusion Criteria*	
1) Personal history of ovarian cancer	
2) Unable to speak English	
3) Unable to provide informed consent	
4) Unwilling to complete baseline and follow-up questionnaires	
5) Unable to access the internet	
6) Previous genetic testing or counseling regarding cancer risk	
7) Previous bone marrow transplant	
8) Previous blood transfusion (7 days prior to genetic testing)	
9) Active hematologic malignancy	
10) Residents of the state of New York	

Recruiting efforts are being carried out through clinical settings, advocacy groups, and various social media platforms. The different recruitment strategies were designed to capture a heterogeneous group of women, which will allow us to assess whether we improve access to genetic testing in diverse patient populations stratified for race and ethnicity, as well as, socioeconomic, educational and geographical backgrounds.

### Remote consenting process

Given the size and large geographic area (all 50 U.S. states and the District of Colombia) for which we are recruiting and enrolling participants, the use of electronic informed consent (IC) became a vital component to the study. Electronic informed consent is defined by the FDA as the use of electronic systems and processes that may employ multiple electronic media, such as text, graphics and videos, to convey information related to the study and obtain and document informed consent (http://www.fda.gov/downloads/drugs/guidancecomplianceregulatoryinformation/guidances/ucm436811.pdf).

Our biggest challenge was ensuring that the person signing the IC is the same as the person completing the study procedures. We accomplished this by requiring all participants to create a unique username and password that must be used to confirm their identity before signing the IC and before completing each study survey. To ensure that consent is truly informed, we created a series of comprehension questions that the participant must answer correctly before being allowed to sign the IC. The IC was delivered to study participants by email using the survey tools in REDCap. All other aspects of the FDA draft guidance were followed, including allowing the participant the ability to print a copy of the IC and call the study coordinator for questions (http://www.fda.gov/downloads/drugs/guidancecomplianceregulatoryinformation/guidances/ucm436811.pdf).

### Measure design

To fully evaluate the efficacy of these alternate models, we are assessing several different outcome measures, including: cancer-risk distress, testing completion rate (defined as receiving results), genetics knowledge, cognitive outcomes (i.e. decisional satisfaction, decisional regret), and behavioral outcomes. Surveys are completed at baseline, 3 months, 12 months and 24 months post-results disclosure. The specific measures and time-points are summarized in Table 3. These measures were informed by the prior work of Kinney et al. and Schwartz et al. [4, 5]. Following the Peshkin et al. method of classifying these measures, these variables were chosen to assess cognitive (decisional regret and decisional satisfaction), affective (distress, anxiety, depression), and behavioral outcomes [18]. Questionnaires for assessing outcome measures are delivered to study participants by email using the survey features of REDCap. Questionnaires are distributed in an automated fashion, based on the date of the participant’s results disclosure.

**Table 3:** Variables, Measures and Time Points

TIMEPOINTS					
Outcome Variables	Measures	Baseline	3 months post-results disclosure	12 Months post-results disclosure	24 Months post-results disclosure
Cancer Risk Distress	Impact of Events Scale (IES) [9]	X	X	X	X
Decisional Satisfaction Concerns	Satisfaction with Decision (SWD)[10]		X	X	X
Genetic Testing Concerns	Multidimensional Impact of Cancer Risk Assessment (MICRA) [11]		X	X	X
Anxiety	Generalized Anxiety Disorder Scale (GAD-7) [12]	X	X	X	X
Depression	Patient Health Questionnaire (PHQ-8)[13]	X	X	X	X
Decisional Regret	Decision Regret Scale [14]		X	X	X
Quality of Life	Veterans RAND 12-Item Survey (VR-12) [15]	X	X	X	X
Surveillance Behaviors	Behavioral Risk Surveillance Study Measures [16]	X	X	X	X
Genetics Knowledge	General Knowledge about HBOC [17]	X	X	X	X
Cancer Risk Distress	Demographics	X			

The primary study outcome is cancer-risk distress at 3 months post-result disclosure, as measured by the Impact of Events Scale (IES) [9, 19].

### Study interventions

All participants receive online genetic education, including a 5-min pre-test educational video and an online results report. The video provides an explanation of hereditary cancer, inheritance patterns, associated cancer risks, and an overview of the possible results. Participants randomized to receive pre-test telephone counseling are prompted to schedule their telephone appointments online and will complete telephone counseling in addition to the online genetic education. The usual content of pre- and post-test genetic counseling for hereditary cancer risk has been well described [20, 21]. Following these standard guidelines, a genetic counseling outline was created by the study team and is followed by the board-certified genetic counselors involved in the study to guarantee consistency in counseling and ensure standardization for the purposes of the study (Table 4).

**Table 4:** Genetic Counseling Outline

Pre-Test Genetic Counseling	
• Review/verification of the family history provided in the online survey	
• Review the basics of hereditary cancer *(incidence, inheritance pattern, define genes & mutations)*	
• Review the benefits and limitations of genetic testing	
• Briefly review the cancer risks	
• Review result possibilities and their implications	
• Briefly discuss the benefits and limitations of GINA	
• Confirm that participant would like to move forward with testing	
• Assess for distress throughout the session using psychosocial training.	
• Documentation of the session and amount of time spent on the phone	
Post-Test Genetic Counseling	
• Verify the participant’s continued interest in receiving genetic test results and encourage them to follow along with their results online	
• Provide genetic test results	
• Review implications of test results for the participant and family members	
• Assess the response of individual to the information provided	
• Allow time for questions or voicing of concerns	
• Provide phone number for contact if needed and information for local GC	
• Assess for distress throughout the session using psychosocial training.	
• Document the session and the amount of time spent on the phone	

### Statistical considerations

We employ a non-inferiority design with a non-inferiority margin of 4 points, as suggested by Schwarz et al. (2014), for the difference in IES score between the control arm and each of the other three study arms at 3 months post-result disclosure.

We are enrolling 5200 eligible women with a goal of 3000 completing genetic testing. We expect that around 85% (*n* = 4400) of those who consent to participate will complete the baseline questionnaire and then be randomized. Of these, we will enroll around 750 subjects with a known familial mutation into the cascade group, with the remaining 3650 subjects, who have an increased likelihood of carrying a deleterious mutation in an ovarian cancer predisposition gene based on personal and family history of disease, as the group of interest for our primary analysis. We expect that 65% (*n* = 2375) of the 3650 participants with personal and/or family history of disease will follow through with providing a saliva sample for analysis, with 75% of those subjects completing the study through the 3 month assessment (*n* = 1780), which equates to about 450 participants on each of the four study arms.

Each of the three experimental study arms will be compared to the control study arm using a one-sided t-test, with the null hypothesis for each of the three tests that the mean stress score in the experimental arm is more than 4 points above the mean stress score in the control arm. A one-sided significance level of 0.025 is targeted across all three tests, resulting in a Bonferroni-corrected significance level of 0.0083 for each test. The sample size was chosen to achieve 93% power for each test, assuming a standard deviation in the scores of 15.3 based on Schwartz et al., yielding an overall power of 80%.

Our secondary outcome is the “completion rate”, defined as a participant progressing through the entire process from pre-test genetic education/counseling to receiving their test results and post-test genetic education/counseling. We will test two null hypotheses regarding the completion rate: that the completion rate for the participants receiving only online genetic education before testing (Arms A and B) is more than 6% less than it is for participants who were randomized to receive telephone genetic counseling before testing (Arms C and D), and that the completion rate for the participants receiving only online genetic education after testing (Arms A and D) is more than 6% less than it is for participants who were randomized to receive telephone genetic counseling after testing (Arms B and D). This 6% non-inferiority margin is more conservative than the 7.5% margin used by Schwartz et al. [5].

In addition to testing our primary and secondary hypotheses, we will summarize for each study arm the key outcomes related to cognition, affect and behavior, and the changes in scores from baseline. We will also use regression models to model the scores (and changes in the scores from baseline) as a function of study arm, risk group, and other covariates.

## Discussion

In-person genetic counseling is likely to remain standard practice in large cancer centers and academic institutions. However, alternative service delivery models for genetic counseling must be further explored to find an optimal balance of clinical quality with increased access and financial sustainability. This is especially important given the largely debated topic of population screening for *BRCA1, BRCA2,* and Lynch syndrome. The discussions surrounding population-based screening for hereditary cancer genes has highlighted a challenge regarding the ability to provide this service on a large scale in an efficient and effective manner [22]. Determining the efficacy of an online genetic SDM may play a vital role in future implementations of population screening.

The nationwide MAGENTA trial is the first study to assess the use of a genetic service model using online access and electronic education for population based testing in individuals at elevated risk of ovarian cancer, while evaluating the need for personal pre and post-test genetic counseling. Innovative service delivery models are essential to decreasing barriers to genetic testing, particularly among underserved populations. Identifying more individuals with inherited cancer susceptibility will allow targeted prevention and ultimately save lives.

## Data Availability

Not applicable. The trial is ongoing and no data is available.

## Abbreviations

IC: Informed consent
MAGENTA: Making genetic testing accessible
OC: Ovarian cancer
SDM: Service delivery model
